# DHCR24 exerts neuroprotection upon inflammation-induced neuronal death

**DOI:** 10.1186/s12974-017-0991-6

**Published:** 2017-11-07

**Authors:** Henna Martiskainen, Kaisa M. A. Paldanius, Teemu Natunen, Mari Takalo, Mikael Marttinen, Stina Leskelä, Nadine Huber, Petra Mäkinen, Enni Bertling, Hiramani Dhungana, Mikko Huuskonen, Paavo Honkakoski, Pirta Hotulainen, Kirsi Rilla, Jari Koistinaho, Hilkka Soininen, Tarja Malm, Annakaisa Haapasalo, Mikko Hiltunen

**Affiliations:** 1 Institute of Biomedicine, University of Eastern Finland, P.O. Box 1627, FI-70211 Kuopio, Finland; 2 A.I. Virtanen Institute for Molecular Sciences, University of Eastern Finland, P.O. Box 1627, FI-70211 Kuopio, Finland; 3 School of Pharmacy, University of Eastern Finland, Kuopio, Finland; 4 Minerva Foundation Institute for Medical Research, Helsinki, Finland; 5 Institute of Clinical Medicine – Neurology, University of Eastern Finland, Kuopio, Finland; 6 Department of Neurology, Kuopio University Hospital, Kuopio, Finland

**Keywords:** Alzheimer’s disease, DHCR24/Seladin-1, Neuroinflammation, Neuroprotection

## Abstract

**Background:**

DHCR24, involved in the de novo synthesis of cholesterol and protection of neuronal cells against different stress conditions, has been shown to be selectively downregulated in neurons of the affected brain areas in Alzheimer’s disease.

**Methods:**

Here, we investigated whether the overexpression of DHCR24 protects neurons against inflammation-induced neuronal death using co-cultures of mouse embryonic primary cortical neurons and BV2 microglial cells upon acute neuroinflammation. Moreover, the effects of DHCR24 overexpression on dendritic spine density and morphology in cultured mature mouse hippocampal neurons and on the outcome measures of ischemia-induced brain damage in vivo in mice were assessed.

**Results:**

Overexpression of DHCR24 reduced the loss of neurons under inflammation elicited by LPS and IFN-γ treatment in co-cultures of mouse neurons and BV2 microglial cells but did not affect the production of neuroinflammatory mediators, total cellular cholesterol levels, or the activity of proteins linked with neuroprotective signaling. Conversely, the levels of post-synaptic cell adhesion protein neuroligin-1 were significantly increased upon the overexpression of DHCR24 in basal growth conditions. Augmentation of DHCR24 also increased the total number of dendritic spines and the proportion of mushroom spines in mature mouse hippocampal neurons. In vivo, overexpression of DHCR24 in striatum reduced the lesion size measured by MRI in a mouse model of transient focal ischemia.

**Conclusions:**

These results suggest that the augmentation of DHCR24 levels provides neuroprotection in acute stress conditions, which lead to neuronal loss in vitro and in vivo.

**Electronic supplementary material:**

The online version of this article (10.1186/s12974-017-0991-6) contains supplementary material, which is available to authorized users.

## Background

Alzheimer’s disease (AD) is a progressive neurodegenerative disorder and the most common form of dementia worldwide. AD is neuropathologically characterized by the deposition of β-amyloid plaques and neurofibrillary tangles in the neocortex of the brain. β-Amyloid plaques are composed of β-amyloid (Aβ) peptides, released from amyloid precursor protein (APP) as a result of sequential proteolytic cleavage by β- (BACE1) and γ-secretases [1]. According to the amyloid cascade hypothesis, impaired balance between the production and clearance leads to accumulation of Aβ, which triggers downstream events in AD pathology, such as formation of neurofibrillary tangles and induction of neuroinflammation and oxidative stress [2].

*DHCR24* (3-β-hydroxysteroid-Δ-24-reductase), also known as seladin-1 (selective Alzheimer’s disease indicator-1), was initially identified as being expressed at lower levels in the affected as compared to unaffected brain regions in AD patients [3, 4]. Thus, it was suggested to represent a selective indicator of AD pathogenesis. More recently, the idea that DHCR24 downregulation would selectively indicate AD pathogenesis [3] has been questioned [5]. On the other hand, genetic polymorphisms in *DHCR24* may modulate the risk of developing AD in humans [6], suggesting that *DHCR24* could be genetically associated with AD. Functionally, DHCR24 protein is characterized as a multifunctional enzyme having two distinctive activities: cholesterol-producing activity, i.e., reduction of desmosterol to cholesterol, mediated by the C-terminal region, and H_2_O_2_-scavenging activity brought about by the oxidoreductase domain near the N-terminus [7, 8]. An accumulating body of evidence strongly suggests that DHCR24 has neuroprotective properties, which might be associated with these activities. DHCR24 has proven protective in different AD-related stress conditions, including Aβ-induced, oxidative, or endoplasmic reticulum (ER) stress [3, 9–13]. Moreover, DHCR24 has a pivotal role in the de novo synthesis of cholesterol. Brain cholesterol, which is fundamental to the synaptic formation and normal functioning of the brain, is decreased during both AD and normal aging [14]. In vitro studies suggest that decreased cholesterol levels could contribute to AD in various ways, including increased inflammatory response and increased production or decreased clearance of Aβ [15–17]. Furthermore, decreased levels of DHCR24 lead to the stabilization of BACE1 and consequently to increased β-amyloidogenic processing of APP under apoptotic conditions in vitro [18]. These findings suggest that augmentation of DHCR24 levels in the affected brain areas might provide a potential therapeutic approach to intervene in AD pathogenesis.

To test if enhanced expression of DHCR24 leads to neuroprotection, we have investigated the effects of DHCR24 overexpression in in vitro and in vivo models upon neuroinflammation. Here, we report for the first time that DHCR24 protects neurons from death upon neuroinflammation induced by lipopolysaccharide (LPS) and interferon γ (IFN-γ) in a neuron-BV2 microglial cell co-culture model. Furthemore, mechanistic elucidation revealed that upon neuroinflammation, the overexpression of DHCR24 did not increase total cellular cholesterol content or APP levels, nor did it affect Akt- or ERK-related neuronal survival pathways or caspase-3 activation in the co-cultures. Importantly, overexpression of DHCR24 increased the total number of dendritic spines and the relative proportion of mushroom spines in mature mouse hippocampal neurons in vitro as well as regionally reduced lesion size in vivo in a mouse model of transient focal cerebral ischemia.

## Methods

### Lentiviral constructs

Human DHCR24 cDNA in pLenti-III-HA vector (pLenti-CMV-h-DHCR24) and empty pLenti-III-HA plasmid (both obtained from Applied Biological Materials, Richmond, BC, Canada) as well as green fluorescent protein (GFP) under the chicken beta actin (CAG) promoter was cloned into lentivirus transfer (HIV) plasmid and was packed into third-generation self-inactivating lentiviral particles in the BioCenter Kuopio National Virus Vector Laboratory in Kuopio, Finland.

### Mouse primary cortical neuron and BV2 cell co-culture and lentivirus-mediated gene transfer

Mouse primary cortical neurons were harvested from 18-embryonic-day-old JAXC57BL/6J mouse pups. Single-cell suspension was prepared from the dissected cortices by trypsin digestion and trituration, and the cells were plated on poly-d-lysine-coated cell culture plates in Neurobasal feeding medium containing 2% B27, 2 mM l-glutamine, 100 U/ml penicillin, and 100 μg/ml streptomycin. Neurobasal feeding medium and B27 are serum-free and thus do not contain cholesterol. Cells were grown in a humidified incubator at 37 °C in 5% CO_2_. After 4 days in vitro (DIV), half of the medium was changed to feed the neurons. On DIV4, DHCR24 and control lentiviral particles were used to infect the cells at multiplicity of infection (MOI) 75. After 24 h of lentiviral infection (DIV5), the medium containing the lentiviral particles was removed. BV2 cells were added to the cultures at 1:5 ratio on DIV5 and let to attach for 2 h, after which the neuroinflammation was induced with 200 ng/ml LPS and 20 ng/ml IFN-γ. The anti-inflammatory cytokine interleukin 10 (IL-10, 50 ng/ml, Peprotech) and the inducible nitric oxide synthase (iNOS) inhibitor 1400 W (20 μM, Tocris) were used as positive controls and were added 1 h after seeding of BV2 cells to the co-cultures. Consequently, after the 1-h pre-treatment with IL-10 and 1400 W, neuroinflammation was induced with 200 ng/ml LPS and 20 ng/ml IFN-γ. Samples were collected on DIV7, 48 h after the induction of neuroinflammation.

### Immunofluorescence microscopy

The neurons and BV2 cells were plated on poly-d-lysine-coated glass coverslips in 48-well cell culture plates. All the following steps were performed at room temperature unless otherwise noted. The cells were fixed in 4% paraformaldehyde for 20 min and permeabilized in ice-cold methanol for 8 min at − 20 °C. Unspecific antibody binding was blocked by incubation in blocking solution (PBS containing 1% bovine serum albumin and 10% normal goat serum) for 20 min. The cells were double-stained with mouse anti-MAP2 (1:2000, Sigma, M9942; neuronal marker) and rat anti-CD11b (1:300, Serotec, MCA74G; microglial cell marker) or rabbit anti-GFAP (1:300, Dako, Z0334; astrocyte marker) primary antibodies at + 4 °C overnight, followed by staining with anti-mouse-Alexa488 (1:500, Invitrogen) and anti-rabbit-Alexa594 (1:500, Invitrogen,) or anti-rat Alexa594 (1:500, Abcam) secondary antibodies for 1 h. Cells without any of the primary antibodies were used as negative controls for background staining. In between the antibody incubations, the cells were washed three times in PBS for 10 min. The nuclei were stained with 4′,6-diamidino-2-phenylindole (DAPI, 1:5000 in PBS, Sigma) for 5 min. The coverslips were mounted on objective slides using GelMount mounting media (Sigma). Cells were imaged using a Zeiss Axio Imager fluorescence microscope at × 10 magnification, and images were prepared using the Zeiss ZEN 2012 program. The number of different cell types (neurons, astrocytes and BV2 cells) in the co-cultures was quantified from five different images taken from three individual wells each in every experiment. All in all, on average 7000 cells were counted in each replicate. Addition of BV2 cells onto the neuronal cultures was observed to decrease the number of neurons by approximately 30% as assessed by the MAP2 immunocytochemistry-based neuronal viability assay (described below). Therefore, the quantified values were normalized for the decrease in neuronal number by dividing with the value of 1.3.

### Protein extraction and western blot analysis

Total protein lysates for Western blot were prepared by lysing the cells in T-PER tissue protein extraction reagent (Thermo Scientific) supplemented with protease and phosphatase inhibitor cocktails (× 100, Thermo Scientific) and centrifuging at 10000×*g* for 10 min. BCA protein assay kit (Thermo Scientific) was used to determine protein concentrations, and 15–30-μg protein samples were separated on NuPAGE 4–12% BisTris gel (Life Technologies) and subsequently blotted onto a PVDF membrane. Following antibodies were used to probe the blots: Akt (1:1000, Cell Signaling Technology), p-Akt recognizing Akt phosphorylated at Ser473 (1:1000, Cell Signaling Technology), APP C-terminus (A8717, 1:2000, Sigma), BACE1 (D10E5, 1:1000, Cell signaling technology), caspase-3 (1:1000, Cell Signaling Technology), DHCR24 (C59D8, 1:800, Cell Signaling Technology), cofilin (D3F9, 1:1000, Cell Signaling Technology), p-cofilin recognizing cofilin phosphorylated at Ser3 (sc-271,921, 1:1000, Santa Cruz Biotechnology, Inc.), ERK 2 recognizing ERK 1 and 2 (1:500, Santa Cruz Biotechnology), p-ERK recognizing ERK 1 and 2 phosphorylated at Tyr204 (1:500, Santa Cruz Biotechnology), CREB (1:1000, Cell Signaling Technology), p-CREB recognizing CREB phosphorylated at Ser133 (1:1000, Cell Signaling Technology), neuroligin-1 (sc-365,110, 1:500, Santa Cruz Biotechnology, Inc.), GAPDH (1:15,000, Abcam), and β-actin (1:1000, Abcam). After incubation with appropriate species-specific horseradish peroxidase (HRP)-linked secondary antibodies (GE Healthcare), the proteins were detected using enhanced chemiluminescence substrates (GE Healthcare) and RT ECL Imager (GE Healthcare) or G:BOX Chemi XRQ (Syngene). Quantity One software (Bio-Rad) was used to quantify the protein levels.

### RNA extraction and real-time quantitative PCR analysis

RNA was extracted in TRI reagent following manufacturer’s protocol for RNA isolation. cDNA synthesis was carried out using SuperScript III First-Strand Synthesis System for RT PCR (Life Technologies). Target specific PCR primers for mouse *DHCR24* (5′-CAAGCCGTGGTTCTTTAAGC-3′ and 5′-CATCCAGCCAAAGAGGTAGC), mouse TNFα (5′-CGAGTGACAAGCCTGTAGCC-3′ and 5′-GTGGGTGAGGAGCACGTAGT-3′), mouse BDNF (5′-TGGCTGACACTTTTGAGCAC-3′ and 5′-GTTTGCGGCATCCAGGTAAT-3′), mouse NQO1 (5′-TAGCCTGTAGCCAGCCCTAA-3′ and 5′-GCCTCCTTCATGGCGTAGTT-3′), mouse HMOX1 (5′-GTCAGGTGTCCAGAGAAGGC-3′ and 5′-GCGTGCAAGGGATGATTTCC-3′), and mouse *GAPDH* (5′-AACTTTGGCATTGTGGAAGG-3′ and 5′-ACACATTGGGGGTAGGAACA-3′) were obtained from TAG Copenhagen. FastStart SYBR Green Master (Roche) was used for qPCR. The comparative ΔΔCt method was used to calculate *GAPDH*-normalized expression levels of the target mRNAs.

### Lipid extraction and total cholesterol assay

To extract lipids for the total cholesterol measurement, cells were homogenized in chloroform:isopropanol:Igepal (7:11:0.1) mix and centrifuged for 10 min at 15000×*g*. Supernatant was taken to a clean tube and air-dried at 50 °C and put in a vacuum desiccator for 5 min. Fluorometric Total Cholesterol Assay Kit (Cell Biolabs) was used to measure the total cholesterol levels. The dried lipids obtained from lipid extraction were dissolved in 200 μl assay diluent, and 50 μl dissolved lipid sample was subjected for the analysis. Cholesterol assay was performed according to the manufacturer’s instructions, and the fluorescence signal was measured with excitation wavelength at 560 nm and emission wavelength at 590 nm using Fluorstar Galaxy plate reader. Total cholesterol levels were normalized to total protein levels of each sample.

### Neuronal viability assay

Neuronal viability in the mouse primary cortical neuron and BV2 microglial co-cultures was assessed as described earlier [19]. Briefly, the cells were fixed in 4% paraformaldehyde in PBS for 20 min and then incubated in 0.3% H_2_O_2_ in methanol to permeabilize the cells and block endogenous peroxidase activity. Incubation in blocking solution containing 1% bovine serum albumin and 10% horse serum for 20 min was used to prevent non-specific staining. Neurons were stained by incubation with mouse anti-MAP2 primary antibody (1:2000, Sigma) overnight at + 4 °C. Next, the cells were incubated with biotinylated horse anti-mouse secondary antibody (1:500, Vector labs) for 1 h and ExtrAvidin-HRP (1:500, Sigma) for another 1 h. The cells were washed three times for 10 min in PBS between the antibody incubations. All antibody dilutions were prepared in the blocking solution. Finally, ABTS Peroxidase Substrate solution (Vector Labs) was prepared following the manufacturer’s instructions and added onto the cells. The absorbance was measured using a microtiter plate reader (ELx808, BioTek or Infinite M200, Tecan) at 405 nm and was directly proportional to the number of neurons in the wells. Six replicate wells per assay were measured for each treatment. The measured background absorbance from co-cultures incubated without the anti-MAP2 primary antibody (negative controls, *n* = 6 per assay) were averaged and subtracted from the absorbances in the other wells. The absorbance in the negative control wells was similar to the absorbance measured from wells without any cells. Due to the neuronal loss induced by neuroinflammation, the mean neuronal viability obtained for each group in each experiment was used to normalize BACE1 and Aβ40 levels.

### Aβ, TNFα, NO, and ROS measurements

Conditioned media from the cell samples were collected immediately before protein extraction and centrifuged at 10000×*g* for 10 min. Aβ40 levels in the conditioned media were determined with monoclonal and HRP-conjugated antibody-based Human/Rat β amyloid 40 ELISA kit (Wako, Osaka, Japan). Aβ40 concentrations were normalized to neuronal viability. Mouse TNF alpha ELISA Ready-SET-Go! kit (Affymetrix, San Diego, CA, USA) was used for the detection of tumor necrosis factor α (TNFα) in the conditioned media. Nitric oxide (NO) levels were determined using Griess Reagent Kit for Nitrite Determination (G-7921, Life Technologies) and normalized to neuronal viability determined by the MAP2-ABTS assay described above. All kits were used as instructed by the manufacturers. Reactive oxygen species (ROS) levels in the co-cultures were measured using fluorogenic probe 2′, 7′-Dichlorodihydrofluorescin diacetate (DCFH-DA, Sigma D6883). One hour after adding BV2 cells, samples were labeled with 120 μM DCFH-DA for 30 min. Two hours after adding the BV2 cells, 5 h-neuroinflammation treatment was started. Subsequently, cells were lysed using T-PER lysis buffer (Thermo Scientific), and fluorescence was measured using plate reader at 480 nm/530 nm.

### Mouse primary hippocampal neuron culture, transient transfection, and spine morphology analysis

Primary hippocampal neuronal cultures were prepared from 18-day-old mouse JAXC57BL/6J embryos according to the protocol previously described [20]. Briefly, single-cell solution (240,000 cells/cm^2^) was plated on 8-well chamber slides (LabTek) coated with poly-d-lysine and 30 μg/ml laminin in feeding media composed of Neurobasal medium supplemented with 2% B27, 0.5 mM l-glutamine, 100 U/ml penicillin, and 100 μg/ml streptomycin. Hippocampal neurons were grown in a cell culture incubator at 37 °C in 5% CO_2_. Half of the culture media was replaced with fresh feeding media after every 5 days in vitro. On DIV19, mature hippocampal neurons in 8-well chamber slides were transiently co-transfected with a mixture containing 2 μl of Lipofectamine 2000 (Invitrogen) and 0.3 μg of control plasmid DNA (pLenti-III-HA) or pLenti-CMV-h-DHCR24 and 0.3 μg enhanced green fluorescent protein (pEGFP). Hippocampal neurons were fixed in 4% paraformaldehyde 24 h after transfection. Anti-DHCR24 antibody (C59D8, 1:100, Cell Signaling Technology) was used for immunofluorescence staining of DHCR24 in the hippocampal neurons. Hippocampal dendritic spines from GFP-positive neurons were imaged with a Zeiss Axio Observer.Z1 inverted microscope (63× NA 1.4 oil objective) equipped with Zeiss LSM 800 confocal module (Carl Zeiss Microimaging GmbH, Jena, Germany). Serial Z-stacks of optical sections from dendritic segments were captured for spine analysis performed with NeuronStudio software [21] as described previously [22].

### Animals

Animal experimentation was carried out in accordance with the national regulation and the Council of Europe (Directive 86/609) of the usage and welfare of laboratory animals. Experiments were approved by the Animal Experiment Board of Finland. The JAXC57BL/6J male mice were kept at the National Laboratory Animal Centre at the University of Eastern Finland in a room equipped with 12-h light/dark cycle and controlled humidity. The mice were provided standard laboratory animal chow and water ad libitum. All experiments were carried out during the day light.

### Injection of viral vectors and stroke surgeries

Total of 27 mice with the age of 3 months were randomized into following treatment groups: (1) lenti-GFP injected sham mice (*n* = 7), (2) lenti-GFP injected ischemic mice (*n* = 11), and (3) lenti-DHCR24 injected ischemic mice (*n* = 9). The mice received lenti-GFP or lenti-DHCR24 injections as described earlier with minor modifications [23]. Shortly, the anesthesia was induced using 5% isoflurane, and the mice were attached to the stereotactic frame (David Kopf Instruments, Tujunga, CA, USA). Surgical anesthesia was maintained using 1.8% isoflurane. Core body temperature was maintained at 36.5 ± 0.5 °C using a homeothermic unit (PanLab, Harvard Apparatus, Barcelona, Spain) connected to a rectal probe. An incision was made on the skin above the injection site, and the skull was exposed. Two microliters of the lenti-DHCR24 or lenti-GFP with the titer of 9.19 × 10^9^ TU/ml and 1.87 × 10^9^ TU/ml, respectively, was injected unilaterally into the following coordinates: + 1.8 mm medial/lateral, 0.4 mm anterior/posterior, and − 2.9 mm dorsal/ventral from the bregma. Viruses were injected with a speed of 0.5 μl/min over a period of 4 min using a Hamilton syringe (Hamilton, NV, USA). After the withdrawal of the needle, the skin was sutured and the mice were allowed to wake up. Buprenorphine 0.03 mg/kg, i.p. (Temgesic 0.3 mg/ml), was used as analgesic and was given immediately after the procedure. Four weeks after the injection of the viral vectors, the mice were subjected to transient occlusion of the middle cerebral artery (tMCAO) or sham surgery as described previously [24]. Briefly, the mice were anesthetized with isoflurane as described above. Silicon-coated monofilament with a diameter of 0.21 ± 0.02 mm (Doccol, MA, USA) was inserted through external carotid artery, advanced through the internal carotid artery to occlude middle cerebral artery. After 45 min of occlusion, the filament was withdrawn and the mice received 1 ml of saline subcutaneously. Mice were then allowed to recover in a heating chamber for 60 min and returned to home cage kept on the heating pad for 24 h. Only half of the cage was placed on heating pad to allow mice to choose their environment. Sham mice underwent similar procedure except that the filament was inserted and withdrawn immediately. Total of six mice died after the surgery from the following treatment groups: lenti-GFP injected ischemic mice (*n* = 3) and lenti-DHCR24 injected ischemic mice (*n* = 3). In addition, one mouse was excluded due to unsuccessful occlusion.

### Magnetic resonance imaging and quantification of lesion volumes

Magnetic resonance imaging (MRI) was performed in anesthetized mice at 1 day post-injury for determination of the lesion volume. MRI was carried out using a horizontal 9.4 T (Agilent technologies, CA, USA) interfaced with Agilent Direct Drive console as previously described [25]. Multi-slice T2-weighted images were acquired with echo time/repetition time of 40 ms/3000 ms, matrix size 128 × 256, field of view 19.2 × 19.2 mm^2^, slice thickness 0.8 mm, and number of slices 12. Images were analyzed blinded to the study groups using Aedes software (Kuopio, Finland) running under MatLab program (Math-works, Natick, USA). The lesion volume was calculated using the formula previously described in [26]. Specifically, the infarct volumes were obtained by multiplying the pixel size by the slice thickness. The infarct volumes were quantified using the following formula: Infarct volume = [volume of left hemisphere − (volume of right hemisphere − measured infarct volume)]/volume of left hemisphere [26]. In addition, edema was calculated using the following formula: Edema = (volume of right hemisphere − volume of left hemisphere)/volume of left hemisphere. Both infarct volume and edema were expressed as percentages. The quantification was done by a researcher blinded to the study groups.

### Statistical analysis

Statistical significance between groups was tested using independent samples *t* test or Mann-Whitney *U* test (two groups) and one-way ANOVA followed by LSD post-test or Kruskal-Wallis test followed by pairwise comparisons with Mann-Whitney *U* test (three or more groups) depending on whether the data fulfilled the assumptions for parametric tests. Statistical significance level was set at *p* < 0.05. All statistical analyses were carried out using IBM SPSS Statistics 21.0.

## Results

### DHCR24 overexpression increases neuronal viability in mouse primary cortical neuron-BV2 microglial co-cultures upon neuroinflammation

**Fig. 1:**
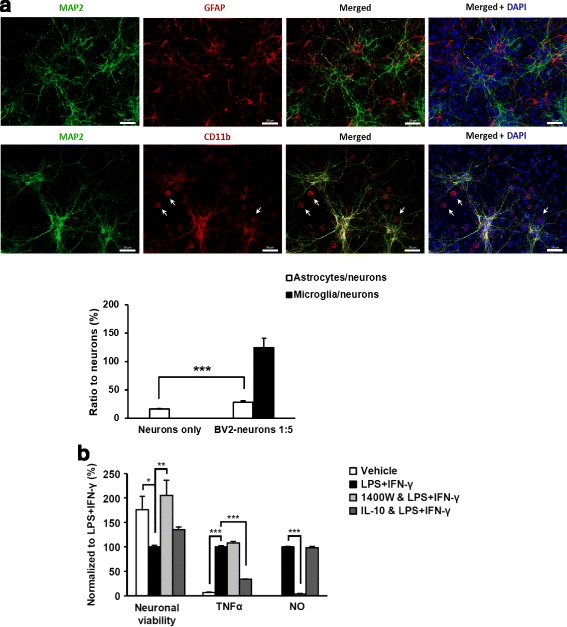
Characterization of the mouse primary cortical neuron and BV2 microglia co-cultures and effects of neuroinflammation. **a** Immunofluorescence staining showed healthy MAP2-positive cortical DIV7 neurons in BV2 microglia co-cultures. Upper panel shows MAP2-positive (green) neurons and GFAP-positive (red) astrocytes. Lower panel shows CD11b-positive (red) BV2 microglia cells (white arrow) in MAP2-positive cortical neuron (green) and BV2 cell co-culture. Nuclei are stained with DAPI. Magnification × 20, scale bar 50 μm. The quantification of the data shows that the ratio of astrocytes to neurons (white column) is significantly increased in the co-cultures compared to neurons only cultures. This might reflect either a true increase in the amount of astrocytes or decreased amount of neurons due to addition of microglial cells. Black column indicates the ratio of BV2 microglial cells to neurons at DIV7. **b** LPS and IFN-γ treatment was used to induce neuroinflammation in mouse primary cortical neuron and BV2 microglia co-cultures. Neuronal viability decreased by 60% after induction of neuroinflammation. Pretreatment with iNOS inhibitor 1400 W (20 μM) prevented neuronal loss and production of NO. Anti-inflammatory cytokine IL10 decreased TNFα levels, but had no effect on viability or NO levels. *p < 0.05, **p < 0.01, ***p < 0.001, mean ± SEM, *n* = 23 (**a**), and *n* = 5–6 (**b**)

Neuroprotective effects of DHCR24 have been extensively studied in the context of several stress conditions, including oxidative stress, ER stress, and apoptosis [3, 9–13, 18]. However, the role of DHCR24 in neuroinflammation, which is a key feature in several neurodegenerative diseases, has not been assessed previously. For this purpose, we utilized co-cultures prepared from embryonic mouse primary cortical neurons and mouse BV2 microglial cells, which were treated with LPS and IFN-γ to induce neuroinflammation at 5 days in vitro according to [19]. Exponentially dividing BV2 microglial cells were added to the E18 cortical cultures after 5 DIV at the ratio of 1:5. Immunofluorescence analysis of co-cultures at 7 DIV showed the expected number of BV2-microglial cells as detected by CD11b staining (Fig. 1a). Interestingly, the number of astrocytes, as detected by GFAP staining, at 7 DIV was significantly increased by approximately 2.5-fold (*p* < 0.001) after the addition of BV2-microglial cells to the cortical cultures (Fig. 1a). Next, we assessed the effect of LPS and IFN-γ specifically on neuronal viability using MAP2-ABTS assay according to [19] and observed an approximately 40% reduction in the neuronal viability after LPS and IFN-γ treatment as compared to vehicle-treated co-cultures at DIV7. The reduction in neuronal viability was accompanied with significantly increased levels of the proinflammatory cytokine TNFα and NO (Fig. 1b). To validate the neuroinflammation model, we pre-treated co-cultures with an anti-inflammatory cytokine interleukin-10 (IL-10) and with a selective inhibitor of inducible nitric oxide synthase (iNOS; 1400 W) to test whether these pre-treatments affect the inflammation response and neuronal viability. Pre-treatment with IL-10 decreased the TNFα levels in LPS- and IFN-γ-treated cells by 60%, whereas it did not affect the neuronal viability. Pre-treatment with the iNOS inhibitor 1400 W, on the other hand, resulted in an average 90% reduction in the NO, but not in the TNFα levels. The 1400 W treatment also restored the neuronal viability to similar level as in the vehicle-treated samples (Fig. 1b). LPS and IFN-γ treatment of mouse primary cortical neuronal cultures without addition of BV2 cells did not affect production of TNFα or NO, or neuronal viability as compared to vehicle-treated samples (data not shown). Collectively, these results are consistent with previous findings in the neuron-BV2 co-culture model upon neuroinflammation [19].

**Fig. 2:**
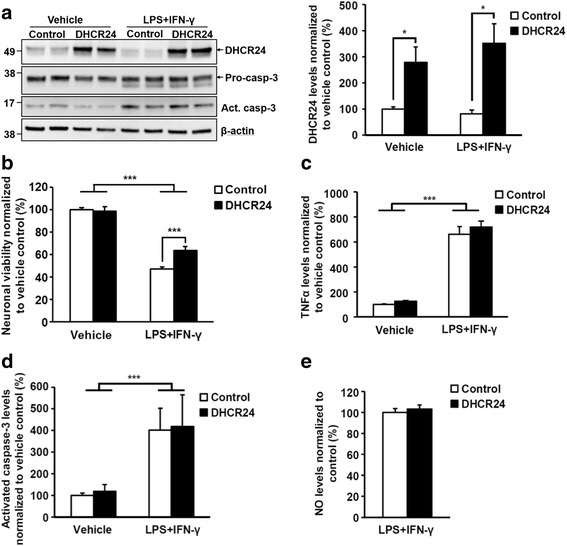
DHCR24 overexpression increases neuronal viability without affecting TNFα, caspase-3, or NO levels in the neuron-BV2 cell co-cultures. **a** DHCR24 levels were significantly increased after lentivirus-mediated gene transfer. Molecular masses in kilodaltons are indicated on the left side of the blots. **b** Viability of mouse primary cortical neurons during LPS and IFN-γ treatment is significantly increased by DHCR24 overexpression. The viability of vehicle-treated neurons overexpressing DHCR24 does not differ from the control cells. **c** TNFα levels were significantly increased after LPS and IFN-γ treatment but unaffected by DHCR24 overexpression. **d** Pro-caspase-3-normalized activated caspase-3 levels were significantly upregulated after LPS and IFN-γ treatment, whereas DHCR24 overexpression had no effect on caspase-3 activation. Western blot of caspase-3 is shown in panel **a**. **e** NO levels did not significantly differ between DHCR24 overexpressing and control cells. NO levels in the vehicle-treated cells were undetectable. *p < 0.05, **p < 0.01, ***p < 0.001, mean ± SEM, *n* = 6 (**a**, **b**, **d**), and *n* = 15–17 (**c**, **e**)

Next, we used lentivirus-mediated gene transduction to overexpress human DHCR24 in the neurons at DIV4 prior to the addition of BV2 microglial cells at DIV5. Subsequently, the neuron-specific expression of exogenous DHCR24 resulted on average in threefold increase in DHCR24 protein levels at DIV7 (Fig. 2a). Endogenous or exogenous DHCR24 expression was not altered by the induction of neuroinflammation (Fig. 2a). Western blot-based analysis of BV2 microglial cells alone with DHCR24-specific antibody revealed only low levels of DHCR24, suggesting that the main portion of endogenous DHCR24 in the co-cultures derives from the neurons (data not shown). Treatment of the co-cultures with LPS and IFN-γ decreased the neuronal viability as expected based on the initial characterization of the co-culture model. Importantly, the overexpression of DHCR24 significantly increased the viability of neurons in LPS- and IFN-γ-treated co-cultures as compared to co-cultures transduced with the control lentivirus (Fig. 2b). However, overexpression of DHCR24 did not affect the production of TNFα or NO, nor the activation of caspase-3 in the co-cultures, which were all upregulated upon LPS- and IFN-γ-induced neuroinflammation (Fig. 2c–e). We also attempted to measure ROS levels 6 h after the induction of neuroinflammation, but the ROS levels were below detection level (data not shown). Collectively, these results suggest that DHCR24 exerts neuroprotection in neuron-BV2 cell co-cultures upon LPS- and IFN-γ-induced neuroinflammation. However, it appears that the underlying neuroprotective mechanism(s) are not directly linked to reduced inflammatory or apoptotic responses, or decreased iNOS activity.

### DHCR24 overexpression does not affect the levels of total cholesterol but increases significantly the levels of neuroligin-1 in mouse primary cortical neuron-BV2 microglial co-cultures upon basal conditions

**Fig. 3:**
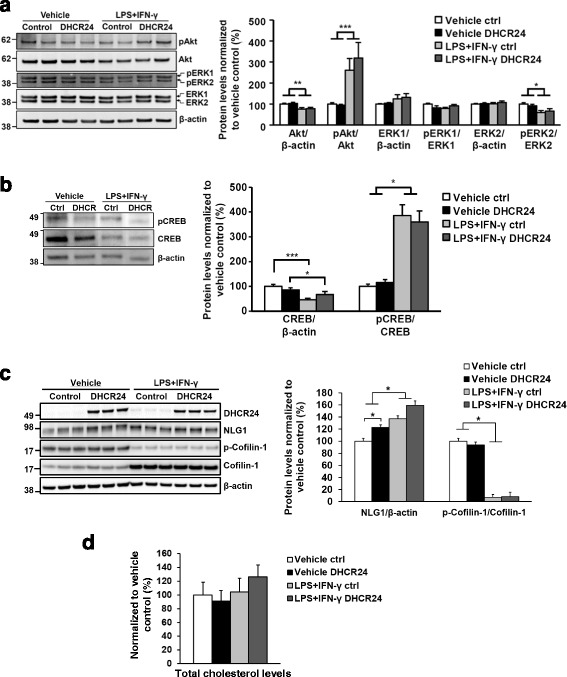
DHCR24 overexpression does not affect the levels of total cholesterol, but increases significantly the levels of NLG1 in the neuron-BV2 cell co-cultures. **a** Western blotting was used to detect the levels of total and phosphorylated forms of Akt, ERK1, and ERK2. DHCR24 overexpression had no significant effect on the levels of these proteins. However, the total Akt levels decreased, and phosphorylated Akt levels increased upon LPS and IFN-γ treatment. Phosphorylated ERK2 levels were significantly reduced after LPS and IFN-γ treatment. **b** Levels of total CREB are decreased in both control and DHCR24-overexpressing co-cultures upon LPS and IFN-γ treatment. Phosphorylation of CREB is increased upon LPS and IFN-γ treatment. Western blot samples in the figure are from the same membrane. **c** The overexpression of DHCR24 significantly increased NLG1 levels upon basal growth conditions in primary neuron-BV2 cell co-cultures. A similar trend towards increase was also observed in co-cultures overexpressing DHCR24 upon LPS and IFN-γ treatment. The assessment of phosphorylation status of cofilin-1 at Ser 3 did not show statically significant changes between DHCR24-overexpressing or control co-cultures after vehicle or LPS and IFN-γ treatments. The induction of neuroinflammation per se significantly reduced the phosphorylation cofilin-1 at Ser 3. **d** Total protein-normalized total cholesterol levels in control and DHCR24-overexpressing co-cultures upon vehicle and LPS and IFN-γ treatments do not show statistically significant changes between groups. Vehicle control was normalized to 100%. *p < 0.05, **p < 0.01, ***p < 0.001, mean ± SEM, *n* = 6 (**a**, **b**), *n* = 3 (**c**), and *n* = 7 (**d**)

Given the observed neuroprotection by DHCR24 overexpression upon LPS- and IFN-γ-induced neuroinflammation in neuron-BV2 cell co-cultures, we next elucidated the activity of well-established regulatory proteins involved in the survival and growth of neurons, namely Akt, ERK1, and ERK2 [27, 28]. To do this, the phosphorylation status of Ser473 in Akt (pAkt) and Tyr204 in ERK1/2 (pERK1/pERK2) needed for the full activation of these kinases was assessed (Fig. 3a). Although the induction of neuroinflammation significantly augmented the phosphorylation of Akt (pAkt/Total Akt), the overexpression of DHCR24 did not modulate the Akt activation. Conversely, the levels of pERK2 were decreased, while pERK1 levels remained unchanged after normalization to the total levels of ERK1/ERK2 upon neuroinflammation (Fig. 3a). The activation of ERK1 or ERK2 was not affected by the overexpression of DHCR24. To further delineate the molecular mechanism underlying the neuroprotective effect of DHCR24 in neuron-BV2 cell co-cultures, the Ser133 phosphorylation of *cAMP* response element-binding protein (CREB) was evaluated. The binding of activated form of this transcription factor to the cAMP response elements modulates the transcription of the downstream genes involved in neuronal survival [29]. As with Akt, the activation of CREB was observed upon the induction of neuroinflammation in neuron-BV2 cell co-cultures without any statistically significant modulation by the overexpression of DHCR24 (Fig. 3b). Elucidation of neuroligin-1 (NLG1) levels revealed a statistically significant increase in DHCR24 overexpressing neuron-BV2 cell co-cultures upon basal growth conditions. A similar trend (*p* = 0.066) was observed in DHCR24 overexpressing neuron-BV2 cell co-cultures upon neuroinflammation (Fig. 3c). Importantly, since NLG1 regulates the dendritic spines and synaptic plasticity via LIMK1/cofilin-mediated actin reorganization, we next determined the phosphorylation status of cofilin at Ser 3 (p-cofilin), which is a reliable indicator of cofilin activity [30]. Although the assessment of total cofilin-normalized p-cofilin levels revealed a strong reduction upon neuroinflammation as compared to basal growth conditions, the levels of p-cofilin were unaltered in neuron-BV2 co-cultures overexpressing DHCR24 in both basal conditions and under neuroinflammation as compared to control co-cultures (Fig. 3c). Finally, given the enzymatic role of DHCR24 in the production of cholesterol from desmosterol and the evidence that the disruption of cholesterol metabolism modulates synaptic function and survival [31], the total cellular cholesterol levels were measured from the vehicle and LPS/IFN-γ-treated neuron-BV2 cell co-cultures transduced with DHCR24 or control lentiviruses (Fig. 3d). The total cellular cholesterol levels normalized to total protein levels (including both cholesterol esters and free cholesterol) were not statistically increased in the DHCR24-overexpressing co-cultures as compared to control co-cultures upon neuroinflammation (Fig. 3d). These findings altogether suggest that DHCR24 does not exert neuroprotection in the neuron-BV2 cell co-cultures upon neuroinflammation via modulation of the well-established neuronal survival pathways regulated by Akt, ERK1/2, or CREB. Also, the improved neuronal survival upon neuroinflammation does not associate with significantly increased total cholesterol levels, but could be linked to improved synaptic plasticity via increased protein levels of NLG1 upon overexpression of DHCR24.

### DHCR24 overexpression reduces the maturation of APP in mouse primary neuron-BV2 microglia co-cultures upon neuroinflammation

**Fig. 4:**
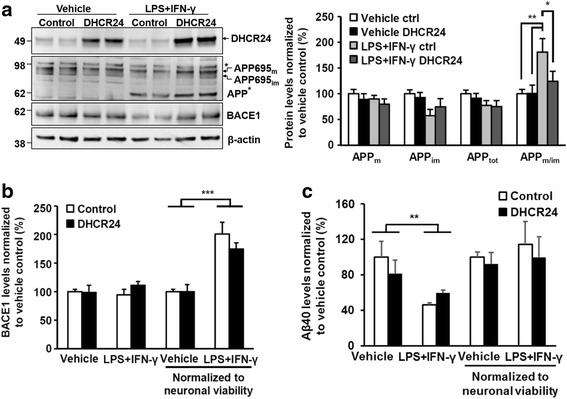
APP, BACE1, and Aβ40 levels are not affected by DHCR24 overexpression or neuroinflammation in the neuron-BV2 cell co-cultures. **a** Western blotting was used to detect the levels of mature (APP695_m_), immature (APP695_im_), and total APP (APP_tot_, calculated as APP695_m_ + APP695_im_). There were no significant differences in the levels of APP_m_, APP_im_, and APP_tot_. However, the ratio between mature and immature APP695 forms was significantly increased in the LPS and IFN-γ-treated control co-cultures. An APP fragment of approximately 60 kDa (APP*) was detected in the LPS and IFN-γ-treated samples, probably resulting from caspase-mediated cleavage of APP. DHCR24 overexpression did not affect the levels of this fragment. Molecular masses in kilodaltons are indicated on the left side of the blots. APP751 isoform derived from glial cells is indicated as with the asterisk (*) above the APP695_m_ and APP695_im_ bands. **b** The levels of BACE1 normalized to neuronal viability are increased upon LPS and IFN-γ treatment, but DHCR24 overexpression has no effect on BACE1 levels. **c** Aβ40 was detected from conditioned media using ELISA. Decreased levels of Aβ40 were detected in the LPS and IFN-γ-treated samples. This was possibly due to neuronal loss in these samples as normalization of Aβ40 levels to neuronal viability abolished the effect. Aβ40 levels were not significantly affected by DHCR24 overexpression. *p < 0.05, **p < 0.01, mean ± SEM, *n* = 6

Keeping in mind the intimate link between cholesterol and APP [32], we next assessed the effects of DHCR24 on APP processing and the generation of Aβ in the neuron-BV2 co-cultures upon LPS- and IFN-γ-induced neuroinflammation (Fig. 4). The induction of neuroinflammation significantly affected the maturation of neuron-specific APP695 isoform by increasing the ratio of APP mature vs. APP immature (APP_m/im_) in the control- but not in the DHCR24-transduced co-cultures (Fig. 4a). Conversely, the total levels or maturation of APP695 isoform remained unaffected by the overexpression of DHCR24 in the co-cultures upon normal growth conditions. A potential caspase-cleaved fragment of APP (> 60 kDa) [33] was detected in the LPS- and IFN-γ-treated samples, but there were no changes in the levels of this fragment between DHCR24- and control-transduced samples (Fig. 4a, data not shown). Finally, the overexpression of DHCR24 did not affect the levels of BACE1 in the total protein lysates or the levels of Aβ40 in the conditioned media (Fig. 4b, c). Given the fact that BACE1 and APP695 isoform are abundantly expressed in the neurons, BACE1 levels were normalized to the neuronal viability in the co-cultures. Although the levels of BACE1 were significantly augmented due to the induction of neuroinflammation, the levels of BACE1 were not significantly altered in co-cultures overexpressing DHCR24 as compared to control samples upon neuroinflammation (Fig. 4b). Similarly, the normalization of Aβ40 levels to neuronal viability did not reveal significant changes between DHCR24- and control-transduced samples (Fig. 4c). These findings suggest that the neuron-specific maturation of APP695 is reduced due to the overexpression of DHCR24 in the mouse primary neuron and BV2 microglia co-cultures upon neuroinflammation, coinciding with the increased neuronal viability.

### The overexpression of DHCR24 increases total dendritic spine and mushroom spine density in mouse hippocampal neurons

**Fig. 5:**
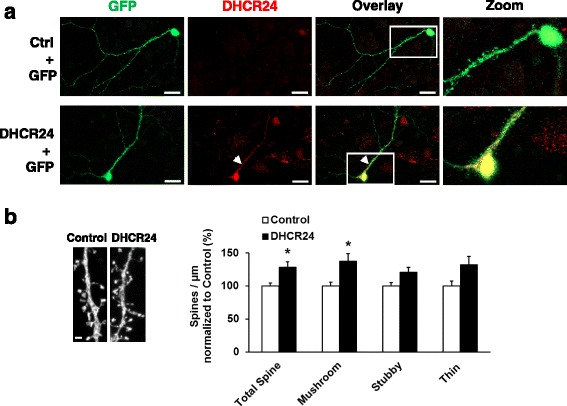
Overexpression of DHCR24 increases total and mushroom spine density in mouse hippocampal neurons. **a** Immunofluorescence staining of mouse hippocampal neurons (DIV20) with DHCR24 antibody. Neurons were transiently transfected with control + GFP plasmids (top row), or DHCR24 + GFP plasmids (lower row), where the white arrow points to the GFP and DHCR24-positive dendrite. Magnification of the GFP-positive cell soma and part of the dendrite inside the white box is shown next to the overlay picture. Magnification × 63, scale bars 20 μm. **b** Representative images of dendritic segments transiently transfected either with control or DHCR24 plasmid. Spine density quantification demonstrates that transient overexpression of DHCR24 significantly increases total spine density and mushroom spine density per micrometer in hippocampal neurons (DIV20). *p < 0.05, mean ± SEM, *n* = 40 (the total number of neurons analyzed from three independent experiments). Magnification 63×, zoom 3×. Scale bar 1 μm

We next assessed the potential effects of DHCR24 on synapses by determining the dendritic spine number and morphology in mature mouse hippocampal neurons (DIV20) upon normal growth conditions. It has been previously shown that the local cholesterol synthesis as well as membrane cholesterol levels are key factors in regulating the development of mature synapses and spine density [34]. Immunofluorescence confocal microscope analysis showed endogenous DHCR24 expression in the soma of GFP plasmid-transfected mouse hippocampal neurons (Fig. 5a, top row). Hippocampal neurons co-transfected with DHCR24 and GFP plasmids (Fig. 5a, bottom row) showed also prominent staining of DHCR24 in the dendrites of the GFP-positive neurons. No immunofluorescence signal was detected in control neurons, which were mock-transfected and incubated only with secondary antibody (data not shown). Consequently, the effects of DHCR24 on the dendritic spine morphology (classified as mushroom, thin, and stubby subtypes), head size, and the total spine number were determined (Fig. 5b). Quantitative analysis revealed that the overexpression of DHCR24 significantly increased the total and mushroom spine density as compared to control plasmid-transfected neurons. The head sizes of the mushroom, thin or stubby spines were not affected by the overexpression of DHCR24 (data not shown). These data suggest that the overexpression of DHCR24 promotes increase in dendritic spine density in mature hippocampal neurons.

### Lentivirus-mediated striatal overexpression of DHCR24 decreases ischemia-induced lesion size

**Fig. 6:**
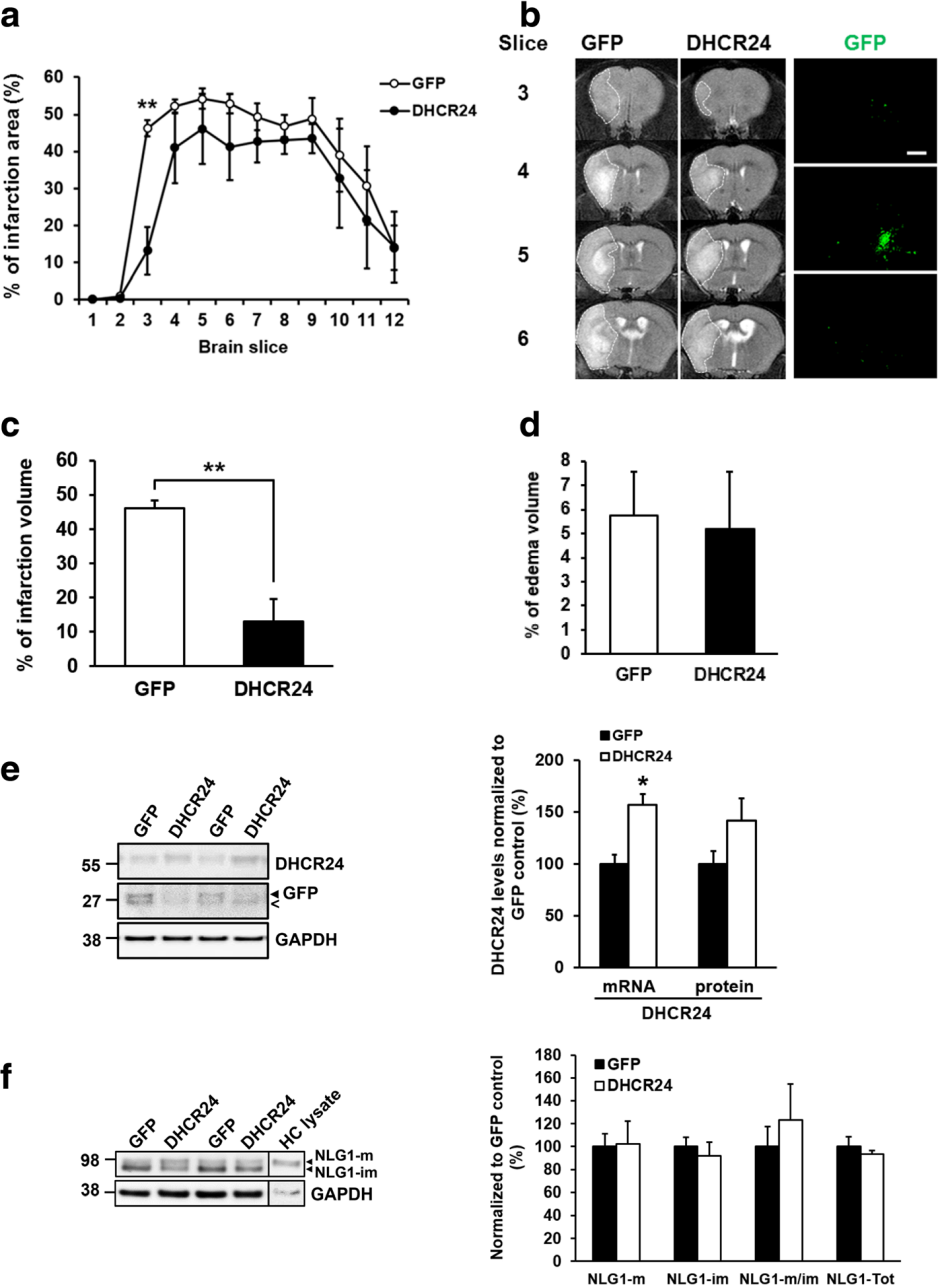
DHCR24 overexpression decreases lesion size after middle cerebral artery occlusion in mice. **a** Lentivirus-mediated overexpression of GFP control or DHCR24 in mouse brain was induced 3 weeks prior to the tMCAO. Lesion size quantification at 1 day after tMCAO from 12 consecutive image planes revealed a significant reduction in the lesion size at the third imaged plane corresponding to the area where the lenti-GFP control (white circle) or lenti-DHCR24 (black circle) injections were done. **b** Representative MRI images of the GFP control and DHCR24 groups. Dotted line surrounds the ischemic lesion in the lenti-GFP control brain. Right panel demonstrates the local expression of GFP after striatal lentiviral vector injection, representative images of 3 coronary brain sections 0.8 mm apart (× 4 magnification, scale bar 200 μM). **c** Quantification data of the third imaged plane in reveals statistically significantly decreased infarction volume in DHCR24-injected animals compared to those injected with GFP control. **d** Lenti-DHCR24 injection had no effect on the extent of edema at the third imaged plane. **e** Western blotting was used to detect the protein levels of DHCR24 and GFP in the whole mouse striatum after lentiviral injection. Molecular masses in kiloDaltons are indicated on the left side of the blots. Less-than sign in the blot picture denotes an unspecific band. Quantification of DHCR24 with qPCR was used to detect the mRNA and Western blot to detect protein levels in the same samples. **f** The levels of mature (NLG1-m), immature (NLG1-im), and total levels (NLG1-m + NLG1-im) of NLG1 were determined from mouse striatum. The whole protein lysates of DHCR24 overexpressing and control samples were analyzed using Western blotting. Total protein lysate from mouse hippocampal primary neuronal cultures (HC) was used as control. In all graphs *p < 0.05, **p < 0.01, mean ± SEM, *n* = 6

In order to elucidate the neuroprotective efficacy of DHCR24 in vivo, C57BL/6J mice were intrastriatally injected with lentiviral vector encoding DHCR24 or GFP (control). Intrastriatal injection of the lentivirus yielded a relatively local overexpression as detected by GFP fluorescence induced by the injection of control lentivector encoding GFP, spanning within approximately 1.6-mm area at the injection site (Fig. 6b). The injected mice were subjected to tMCAO 3 weeks after the injection of lentiviral vectors. Quantification of the MRI images taken at 1 day after stroke revealed very local, yet significant protection against ischemia-induced cell death locally at the DHCR24 lentivirus injection site (Fig. 6a). Figure 6b illustrates typical lesion images of GFP and DHCR24 lentiviral vector injected mice taken at the striatal level close to the lentivirus injection site. The decrease in the lesion size reached statistical significance at the striatal level on the 3rd imaged slice (Fig. 6c), without any effect on the extent of edema (Fig. 6d). Western blot analysis of the protein homogenates from the whole striatum using an antibody against DHCR24 confirmed the increased expression of DHCR24 in the DHCR24 lentivirus-injected ischemic mouse striatum (Fig. 6e). Furthermore, qPCR showed a statistically significant increase in the GAPDH-normalized mRNA levels of DHCR24 in DHCR24 lentivirus-injected ischemic striatal samples as compared to GFP-control lentivirus-injected ischemic striatal RNA-samples (Fig. 6e). In order to elucidate the potential molecular mechanism underlying the reduced lesion size in striatum upon DHCR24 overexpression, the protein levels of NLG1 were determined from the striatal whole cell lysates (Fig. 6f). The total levels of NLG1 were not altered between DHCR24 overexpressing and control samples in striatum. Interestingly, however, maturation of NLG1 (the ratio of mature (m) and immature (im) forms of NLG1; NLG1-m/im) showed a moderate increase in DHCR24 overexpressing mice, but this did not reach statistical significance (Fig. 6f). Finally, we investigated whether the mRNA levels of tumor necrosis factor-α (TNFα), brain-derived growth factor (BDNF), heme oxygenase-1 (HMOX1), and NAD(P)H quinone oxidoreductase-1 (NQO1) are changed in ischemic striatal samples due to the overexpression of DHCR24 using qPCR. TNFα is a marker for inflammation [19], and BDNF is associated with neuronal survival, differentiation and neuronal plasticity [35], while HMOX1 and NQO1 are up-regulated in response to oxidative stress [36, 37]. Consequently, GAPDH-normalized mRNA levels of TNFα, BDNF, HMOX1, or NQO1 did not show statistically significant changes in the ischemic brain tissue upon overexpression of DHCR24 (Additional file 1: Figure S1). Collectively, these data suggest that the overexpression of DHCR24 is able to significantly, yet locally, mitigate the ischemia-induced damage in a mouse model of transient focal cerebral ischemia without significantly affecting the inflammatory, neurotrophic or oxidative stress responses.

## Discussion

DHCR24 has previously been shown to protect neuronal cells in different stress conditions, including oxidative stress, ER stress, Aβ toxicity, and apoptosis [3, 9–13]. Here, we report for the first time that the overexpression of DHCR24 enhances neuronal viability in neuron-BV2 microglial cell co-cultures upon LPS- and IFN-γ-induced neuroinflammation, increases the total number of dendritic spines and the proportion of mushroom spines in mature mouse hippocampal neurons, and is neuroprotective in a mouse experimental model of cerebral stroke. Moreover, our data suggest that the neuroprotection elicited by the overexpression of DHCR24 upon neuroinflammation is not related to significantly altered proinflammatory cytokine response, total cellular cholesterol levels or the activity of proteins linked with neuroprotective signaling, such as Akt, ERK, or CREB. Conversely, the protein levels of NLG1, which is a well-established post-synaptic adhesion protein involved in synaptic plasticity [30, 38, 39], were increased in neuron-BV2 co-cultures upon the overexpression of DHCR24 particularly in the basal growth conditions. These data suggest that yet undefined neuroprotective mechanism(s) may underlie the improved neuronal survival induced by the overexpression of DHCR24 in the used in vitro and in vivo models.

Prior studies have linked the neuroprotective effects of DHCR24 directly to its cholesterol-synthesizing activity, showing cholesterol-dependent protection from oxidative stress [11] or Aβ toxicity [9] and maintenance of lipid raft integrity [10]. Here, a moderate, but statistically non-significant increase in total cellular cholesterol levels was detected, and this coincided with enhanced neuronal survival in the DHCR24-overexpressing neuron-BV2 co-cultures after the induction of neuroinflammation. However, it is possible that the moderately increased total cholesterol levels may simply reflect the increased number of cholesterol-rich neurons rather than increased DHCR24-mediated local synthesis of cholesterol. Also, owing to the fact that only the total cellular cholesterol levels were determined, it is impossible to specifically define whether the overexpression of DHCR24 specifically affected the levels of free cholesterol or cholesterol esters upon neuroinflammation. We also detected increased total and mushroom spine density in the mature mouse hippocampal neurons overexpressing DHCR24 upon basal growth conditions. This is an important finding as dendritic spines are dynamic structures tightly regulated by membrane lipid composition [40, 41]. Thus, it could be hypothesized that a local increase in cholesterol synthesis by transient DHCR24 overexpression may facilitate spine formation in dendrites. Recently, it was suggested that cholesterol levels modulate the NMDA receptor (NMDAR) activity [42], while the activation of NMDAR in turn triggered downstream signaling, promoting either cell survival or cell death [43]. In the present study, the overexpression of DHCR24 did not significantly modulate PI3K/Akt, ERK1/2-mediated MAP kinase, or CREB signaling pathways associated with neuronal survival and growth, suggesting that these pathways do not play a major role in the DHCR24-mediated neuroprotection upon LPS- and IFN-γ-induced neuroinflammation. Conversely, upon basal growth conditions, the overexpression of DHCR24 significantly augmented the protein levels of NLG1 in neuron-BV2 microglial cell co-cultures, and after the induction of neuroinflammation, a similar trend was observed upon the overexpression of DHCR24. This is an important finding given that the augmented levels of NLG1 have been shown to increase the spine and synapse growth in cultured neurons [38, 39], emphasizing the central role of NLG1 in retaining the synaptic plasticity and integrity. Recently, it was further established that NLG1 regulates the dendritic spines and synaptic plasticity via LIMK1/cofilin-mediated actin reorganization [30]. More specifically, it was shown that NLG1 activates LIM-protein kinase (LIMK1), which in turn promotes the activation of cofilin through the phosphorylation of cofilin at Ser 3. Consequently, the Ser 3 phosphorylated form of cofilin promotes actin assembly, which then facilitates spine and synapse formation as well as long-term potentiation. In the present study, however, we did not observe a significant increase in the cofilin phosphorylation at Ser 3 upon overexpression of DHCR24 in neuron-BV2 microglial co-cultures, suggesting that molecular mechanism(s) other than NLG1/LIMK1/cofilin-mediated actin reorganization underlies the observed neuroprotection upon the overexpression of DHCR24 in co-cultures under neuroinflammation.

Increased plasma cholesterol content has been previously shown to inhibit the maturation of APP during cellular aging [44], suggesting that the age-associated alterations in relation to cholesterol content are instrumental for cellular functions. Here, we observed enhanced maturation of the APP695 isoform in the control co-cultures upon the induction of neuroinflammation, while a similar increase was not detected in the DHCR24-overexpressing co-cultures. Importantly, APP maturation changes were evident only in the stress-induced, but not in the basal growth conditions, and were not associated with significantly altered levels of BACE1 or Aβ. Several lines of evidence suggest that cholesterol is a central component regulating synaptic function and plasticity [14, 40]. Cholesterol and sphingolipids are enriched in the membranes of dendritic spines, and alterations in the levels of these lipids modulate spine morphology and synaptic activity via affecting the arrangement of actin cytoskeleton and the trafficking of NMDA receptors [40]. Moreover, it has been shown that the reduced synthesis of total brain cholesterol is a prominent feature in aging individuals and AD patients [45–47]. This is in line with the recent findings showing that constitutive loss of hippocampal cholesterol impairs cognition in old rats through an Akt-mediated molecular mechanism, leading to reduced hippocampal long-term potentiation [41].

In addition to the cholesterol-synthesizing activity, previous studies have demonstrated that DHCR24 has a direct H_2_O_2_-scavenging activity, which is able to protect cells from oxidative stress [3, 11, 12]. Also, DHCR24 overexpression-mediated protection from ER-stress-induced apoptosis was recently linked to decreased ROS levels [12]. Thus, it is possible that the protection from neuroinflammation-induced neuronal death is also linked to ROS-scavenging feature by DHCR24. Here, the measurements of ROS levels 6 h after the induction of neuroinflammation in neuron-BV2 cell co-cultures did not reveal any quantifiable ROS production, suggesting that ROS are not produced in the early phases of neuroinflammation in this model. Neuroprotective effects of DHCR24 have also previously been linked to the reduced activation of caspase-3 [3, 12], and increased caspase-3 activation was observed when DHCR24 was downregulated in the neuroblastoma cells [18]. Here, the neuronal viability was increased in DHCR24-overexpressing neuron-BV2 cell co-cultures, but DHCR24 did not alleviate caspase-3 activation, indicating that the neuroprotective effect of DHCR24 was not mediated via inhibition of caspase-3 activity. This was also reflected by the unaltered levels of caspase-cleaved ~ 60 kDa APP fragment between DHCR24- and control-transduced neuron-BV2 cell co-cultures upon neuroinflammation.

We also observed that the lentivirus-mediated overexpression of DHCR24 in striatum reduced the ischemia-induced lesion size in a mouse model of transient focal ischemia. MRI images taken at 1 day after stroke revealed a very local, yet significant protection against ischemia-induced cell death (infarct volume) at the close proximity of the lentivirus injection site without any significant effect on edema. Although the protection was very local as the overexpression of the construct did not spread through the injected hemisphere, it provides a proof of principle that DHCR24 is able to confer protection also in vivo. Furthermore, it is expected that the local DHRC24 levels are higher near the injection site similar to that seen with GFP, which in turn could explain why the analyses conducted from the whole cell extracts showed a significant increase only in the mRNA levels of DHCR24, but not in the protein levels. Owing to the fact that the increased levels of NLG1 in the neuron-BV2 cell co-cultures upon the overexpression of DHCR24 were observed, a similar assessment of NLG1 was done in mouse striatal brain tissue. The total protein levels of NLG1 were not significantly altered, while the maturation of NLG1 (measured as the increased ratio of NLG1-m vs. NLG1-im) showed a trend towards an increase in the DHCR24-transduced striatal samples. NLG1 is known to be N- and O-glycosylated [48], but the exact role of this maturation process in the context of synaptic plasticity is not well-established. Thus, it remains to be determined whether enhanced maturation of NLG1 is related to neuroprotective functions upon different acute stress conditions. Finally, elucidation of the expression profile of TNFα, BDNF, HMOX1, and NQO1 did not reveal alterations in mice overexpressing DHCR24 as compared to control mice, suggesting that DHCR24 is able to locally mitigate the ischemia-induced damage in a mouse model of transient focal cerebral ischemia without significantly affecting the inflammatory, neurotrophic, or oxidative stress responses. Collectively, the findings in the present study are in line with a previous study showing that DHCR24 exerts cholesterol-dependent neuroprotection in an experimental stroke model in mice, in which DHCR24 was genetically downregulated [49]. More specifically, it was suggested that the underlying molecular mechanism of DHCR24 is linked to the maintenance of lipid raft integrity in astrocytes by assuring the EAAT2-mediated uptake of glutamate excess upon ischemic stress [49]. This is an interesting mechanistic finding also in the context of the present study, as we observed that the number of astrocytes significantly increased after the addition of BV2 microglial cells to the neuronal cultures before the induction of neuroinflammation. Thus, increased expression of DHCR24 also in the astrocytes, and not exclusively in the neurons, might also contribute to the improved neuronal survival upon neuroinflammation in our co-culture model. Taken together, the two in vivo studies conducted in different experimental stroke models reinforce the key role of DHCR24 in molecular processes underlying neuroprotection.

## Conclusions

Collectively, our data indicate that the augmentation of DHCR24 expression is neuroprotective both in vitro and in vivo. Increased levels of DHCR24 protect the neurons during neuroinflammation and brain ischemia and promote synaptic and neuronal health by supporting dendritic spine formation upon basal growth conditions. Together with previous reports, our findings suggest that DHCR24 may exert neuroprotective effects in different stress conditions linked to AD and other neurodegenerative diseases and thus may prove as a useful target when considering new therapeutic strategies against synaptic dysfunction and neurodegeneration.

## Additional file

Additional file: Figure S1. Overexpression of DHCR24 does not significantly alter the mRNA levels of TNFα, BDNF, HMOX1, and NQO1 in the whole mouse striatum after middle cerebral artery occlusion. GAPDH-normalized TNFα, BDNF, HMOX1, and NQO1 mRNA levels in the whole mouse striatum determined using qPCR do not indicate changes in the striatum between DHCR24 overexpressing and control lentivirus-transduced mice after tMCAO. Mean ± SEM, *n* = 6. (PDF 228 kb)

## Abbreviations

AD: Alzheimer’s disease
APP: Amyloid precursor protein
Aβ: β-Amyloid
BDNF: Brain-derived neurotrophic factor
CREB: cAMP response element-binding protein
DCFH-DA: 2′7’-Dichlorodihydrofluorescin diacetate
DHCR24: 3-β-Hydroxysteroid-Δ-24-reductase
DIV: Days in vitro
ER: Endoplasmic reticulum
GFP: Green fluorescent protein
HMOX1: Heme oxygenase-1
HRP: Horseradish peroxidase
IFN-γ: Interferon γ
IL-10: Interleukin-10
LPS: Lipopolysaccharide
MRI: Magnetic resonance imaging
NLG1: Neuroligin-1
NO: Nitric oxide
NQO1: NAD(P)H Quinone Dehydrogenase 1
ROS: Reactive oxygen species
tMCAO: Transient occlusion of middle cerebral artery
TNFα: Tumor necrosis factor α
