# Supplementary figures and images for: DHCR24 exerts neuroprotection upon inflammation-induced neuronal death
Source: J Neuroinflammation. 2017 Nov 7;14:215. doi: 10.1186/s12974-017-0991-6 (PMC5678793; doi:10.1186/s12974-017-0991-6)

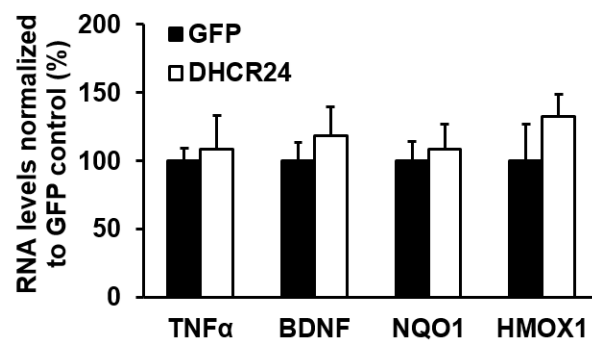

Fig. S1

Supplement: Additional file: Figure S1. — Overexpression of DHCR24 does not significantly alter the mRNA levels of TNFα, BDNF, HMOX1, and NQO1 in the whole mouse striatum after middle cerebral artery occlusion. GAPDH-normalized TNFα, BDNF, HMOX1, and NQO1 mRNA levels in the whole mouse striatum determined using qPCR do not indicate changes in the striatum between DHCR24 overexpressing and control lentivirus-transduced mice after tMCAO. Mean ± SEM, n = 6. (PDF 228 kb) [file 12974_2017_991_MOESM1_ESM.pdf]
